# Favorable Heteroaromatic Thiazole-Based Polyurea Derivatives as Interesting Biologically Active Products

**DOI:** 10.3390/polym15122662

**Published:** 2023-06-13

**Authors:** Mostafa A. Hussien, Gadeer R. Ashour, Soha M. Albukhari, Tamer S. Saleh, Mahmoud A. Hussein

**Affiliations:** 1 Chemistry Department, Faculty of Science, King Abdulaziz University, P.O. Box 80203, Jeddah 21589, Saudi Arabia; 2 Department of Chemistry, Faculty of Applied Sciences, Umm Al Qura University, P.O. Box 24451, Makkah 21955, Saudi Arabia; 3 Chemistry Department, Faculty of Science, University of Jeddah, P.O. Box 80327, Jeddah 21589, Saudi Arabia

**Keywords:** sulfur-containing polyurea, biologically active, heteroaromatic thiazole moiety, thermal properties

## Abstract

This research sought to synthesize a new set of heteroaromatic thiazole-based polyurea derivatives with sulfur links in the polymers’ main chains, which were denoted by the acronyms PU_1–5_. Using pyridine as a solvent, a diphenylsulfide-based aminothiazole monomer (M2) was polymerized via solution polycondensation with varied aromatic, aliphatic, and cyclic diisocyanates. Typical characterization methods were used to confirm the structures of the premonomer, monomer, and fully generated polymers. The XRD results revealed that aromatic-based polymers had higher crystallinity than aliphatic and cyclic derivatives. SEM was used to visualize the surfaces of PU_1_, PU_4_, and PU_5_, revealing spongy and porous shapes, shapes resembling wooden planks and sticks, and shapes resembling coral reefs with floral shapes at various magnifications. The polymers demonstrated thermal stability. The numerical results for PDTmax are listed in the following order, ranked from lowest to highest: PU_1_ < PU_2_ < PU_3_ < PU_5_ < PU_4_. The *FDT* values for the aliphatic-based derivatives (PU_4_ and PU_5_) were lower than those for the aromatic-based ones (616, 655, and 665 °C). PU_3_ showed the greatest inhibitory impact against the bacteria and fungi under investigation. In addition, PU_4_ and PU_5_ demonstrated antifungal activities that, in contrast with the other products, were on the lower end of the spectrum. Furthermore, the intended polymers were also tested for the presence of the proteins 1KNZ, 1JIJ, and 1IYL, which are frequently utilized as model organisms for *E. coli* (Gram-negative bacteria), *S. aureus* (Gram-positive bacteria), and *C. albicans* (fungal pathogens). This study’s findings are consistent with the outcomes of the subjective screening.

## 1. Introduction

Polyurea derivatives are a group of interesting and varied linear polymeric materials that are made using the traditional method of polycondensation. These polymers feature an architecture in the main chain of the polymer akin to that of urea. Similar polymers can also be created by associating diamine compounds with primary amino groups with diisocyanate compounds via step-growth addition polymerization with no by-products [1,2,3,4]. This method does not yield any polymers that are similar to those that were made. A common method for the synthesis of polyurea polymers is the condensation of diisocyanates with dicarboxylic acid chlorides in the presence of dry pyridine or through the cationic ring-opening isomerization method, which additionally allows for the production of a new form of thermally stable polyurea polymerization, as reported by Miyamoto et al. [5]. When sulfur is added to these polymers in any form, or when it is introduced to the monomers, a material called sulfur-containing polyurea is produced. This substance is quite similar to conventional polyurea in terms of both its manufacture and qualities. In recent years, a number of different polymers have garnered significant attention as a result of the large diversity of industrial applications and economic benefits that they offer [3,6]. Due to the extensive degree of intermolecular hydrogen bonding in polyurea, both conventional and sulfur-containing polyurea have high mechanical toughness [1,4,7,8]. Additionally, they have thermal and chemical resistance, which enables them to be used for a wide variety of applications, including in biomedicine, inks, dyes, corrosion protection adhesives, and coatings [5,9,10,11,12,13]. When the full delocalization of electrons in the form of nitrogen, oxygen, and sulfur occurs, the polymers might be deemed to be more corrosion-resistant at lower concentrations. This present study continues the previous research concerning the preparation of different types of organic polymers and/or polymer nanocomposites with interesting features and thus widespread applications in various fields of study [14,15].

Thiazole is a five-membered heteroaryl ring structure that incorporates nitrogen and sulfur atoms, making it a flexible entity in terms of behaviors and responses. The thiazole moiety has been an important heterocycle in the field of chemistry for many decades. The thiazole ring is made up of sulfur and nitrogen, which are arranged so that the pi (π) electrons are allowed to travel from one bond to another, giving the ring aromatic characteristics. In recent decades, the thiazole moiety has garnered a great deal of attention, with numerous review articles emphasizing the importance of the thiazole nucleus in the design and optimization of more bioactive therapeutic candidates [16,17,18,19,20]. Although free thiazole is not naturally accessible, the thiazole ring can indeed be found in peptide alkaloids, metabolites, and cyclopeptides [21]. The lone pair of electrons in the sulfur atom of the thiazole ring is dislocated, satisfying the Huckel rule stipulating the need for at least six pi (π) electrons [22]. Thiazole is subjected to a variety of reactions, including arylation, photochemical reaction, oxidation, intramolecular nucleophilic substitution, dimerization, cycloaddition, donor–acceptor transformation, and others [23,24,25,26,27,28,29]. Our research group has illustrated distinct sulfur-containing classes of polyurea structures, which have been published in the relevant academic literature. In order to limit corrosion, a set of polyureas that have been previously described were synthesized. These polyureas are based on diaryl ether, and the thiazole moiety was incorporated into the main chain of the polymer. When tested for its resistance to corrosion on steel in the presence of 0.5 molar H_2_SO_4_ at 40 degrees Celsius, this particular type of polyurea derivative demonstrated cathodic inhibition [30]. The synthesis of another thiazole-containing polyurea that presents various characteristics through the incorporation of diarylidenecycloalkanone moieties into the main chain of the polymer has been reported. These moieties were included in the polymer. Cathodic, anodic, and mixed inhibitions were observed when the proposed polymers were tested as corrosion inhibitors on steel in the presence of 0.5 M concentrated sulfuric acid at 40 °C [31]. Cathodic, anodic, and mixed inhibitions were observed. More recently, we synthesized a new category of biologically active polyurea carrying the same heterocyclic thiazole moieties along with the compound’s related TiO_2_-doped nanocomposite materials. We also studied the effect of such inclusion on the overall performance of these materials, ranging from identification to the biological screening effect [32]. Variable types of polyureas have been additionally introduced to the literature based on different strategies [33,34,35,36,37,38,39,40,41,42]. As a result, and in accordance with the discussion above, a straightforward polycondensation tool was used to produce another new class of sulfur-containing polyurea derivatives and intriguing heteroaromatic thiazole moieties. To describe the materials that were made, their crystallinity, thermal behavior, solubility, and shapes were studied. In addition, their molecular weights were measured via the GPC of the obtained products. Further, the antimicrobial screening of the targeted materials was investigated against selected Gram-positive, Gram-negative, and fungal strains. All targeted polymers were additionally screened for the *1KNZ*, *1JIJ*, and *1IYL* proteins, which are commonly used as model organisms for *E. coli* (Gram-negative bacteria), *S. aureus* (Gram-positive bacteria), and *C. albicans* (fungal pathogens).

## 2. Experimental Procedures

### 2.1. Measurements

Both monomers’ melting temperatures were measured using a digital image-processing automated melting point device. A Perkin-Elmer Infrared Spectrophotometer was used to obtain Fourier transform infrared spectra (FT-IR). All spectra were collected between wavenumbers of 600 and 4000 cm^−1^. The ^1^H NMR and ^13^C NMR spectra, obtained using CDCl_3_ and DMSO-d_6_, respectively, were recorded on a Bruker Advance 850 MHz spectrometer. The produced polymers’ solubility characteristics were estimated under the same conditions with numerous solvents, namely, dimethylformamide (DMF), dimethyl sulfoxide (DMSO), benzene (C_6_H_6_), chloroform (CHCl_3_), dichloromethane (CH_2_Cl_2_), tetrahydrofuran (THF), acetone, formic acid, and concentrated sulfuric acid. The molecular weights were evaluated using gel permeation chromatography (GPC) on Agilent-GPC. G-1362A was used as the refractive index detector and was operated at 100-104-105 A°. For this experiment, polystyrene was used as a standard, and THF was used to elute the columns at a flow rate of 1 mL min^−1^. Flow rate = 2000 mL min^−1^, injection volume = 100,00 L, and sample concentration = 1.000 g L^−1^ were the operating parameters for the GPC apparatus. Using a RigakuUltima IV X-ray diffractometer, the following settings were applied to a to estimate X-ray diffraction patterns: Ni-filtered Cu K radiation at 40 kV voltage and 40 mA current across a range of 5° to 80° in increments of 0.02° and a sampling speed of 4.0000 deg/min. The TGA thermal performance of the new heteroaromatic thiazole-based polyurea derivatives was displayed using a DTG-60H thermal analyzer. Tests were achieved by placing the samples on a Platinum Macro Pan with an applied heating rate of 10 °C/min within a temperature range of 30–800 °C under a nitrogen atmosphere.

The surface morphology characteristics of the novel heteroaromatic thiazole-based polyurea derivatives were determined via field emission scanning electron microscopy (FESEM) (Jeol JSM-7600F) using a Quanta FEI instrument.

### 2.2. Reagents and Solvents

Diphenylsulfide and chloroacetyl chloride were obtained from Merck and used as received. Sigma-Aldrich was contacted to procure anhydrous aluminum chloride. Thiourea, sodium carbonate, sodium hydroxide, and sodium carbonate anhydrous were all purchased from Fluka. BDH was the source for both acetone and concentrated hydrochloric acid. After their delivery by Merck, 5Å molecular sieves were used to dry carbon disulfide and pyridine. Various diisocyanate compounds (97%) from Sigma-Aldrich were used, including 1,4-phenylenediisocyanate, 4,4′-diphenyl-methanediisocyanate, toluene-2,4-diisocyanate, hexamethylene diisocyanate, and 1,4-cyclohaxylenediisocyanate. Fisher Chemical supplied us with 99.9% ethanol and absolute methanol. BDH was the source for both acetone and concentrated hydrochloric acid. All solvents and reagents were of such high purity (99–97% pure) that they were employed directly after extraction. Absolute ethanol (99%) was obtained from Fisher Chemical. All stated chemicals (solvents and reagents) were utilized exactly as they were purchased, with no additional purification, because of their high purity (99–97%). 

### 2.3. Synthetic Procedures for Monomers and Polymers

#### 2.3.1. Synthesis of 4-Bis-Chloroacetyl-Diphenylsulfide (M1)

A total of 1.59 mL of chloroacetyl chloride (0.002 mol) was dissolved in 50 mL of dry carbon disulfide and poured into 1.6 mL (0.001 mol) of diphenyl sulfide. The mixture was then cooled over an ice bath, and 5.34 g of anhydrous aluminum chloride (0.004 mol) was added dropwise with continuous stirring for 5 h. At the end of the reaction time, all the carbon disulfide had evaporated; then, 60 mL of cold hydrochloric acid was poured into the residue. The resulting product was then filtered, washed with distilled water, and recrystallized, resulting in an orange precipitate with a melting point of 101–103 °C [43].

The FT-IR data of this monomer showed absorption bands at 1580 cm^−1^ for C=C and at 1676 cm^−1^ for the C=O of the chloroacetyl group (Figure S1). ^1^HNMR spectra: (850 MHz, CDCl_3_, δ) = 7.4–7.9 (m, 8 H of aromatic) and 4.6 (s, 4 H of CH_2_choloroacetyl) (Figure S2). ^13^CNMR (850 MHz, CDCl_3_, δ) = 190.24, 141.98, 132.99, 130.82, and 45.76 (Figure S3).

#### 2.3.2. Synthesis of 4-Bis-2-Aminothiazole-Diphenylsulfide (M2)

In a 250 mL round flask attached to a condenser, a mixture of 1 g (0.003 mol) of M1 and 0.47 g (0.006 mol) of thiourea was dissolved in 20 mL of absolute ethanol and refluxed with stirring for 6 h. Then, 25 mL of cold sodium acetate solution (20%; 100 mL) was added to the mixture. The formed precipitate was then collected, filtered, and recrystallized with ethanol, yielding yellowish crystals with a melting point of 240 °C [44]. 

The FT-IR data of this monomer give rise to a band at 1615 cm^−1^, which was attributed to the C=N stretching vibration of the thiazole ring, and two bands were also observed at 3311–3123 cm^−1^, corresponding to the primary amine group (Figure S4). ^1^H NMR spectra: (850 MHz, DMSO-d_6_, δ) = 7.7–7.3 (m, 8 H of aromatic and 2-CH-S) and 6.9 (s, 4 H, NH_2_) (Figure S5). ^13^CNMR (850 MHz, DMSO-d_6_, δ) = 168.77, 149.44, 134.43, 133,79, 131.28, and 127 (Figure S6).

#### 2.3.3. Synthesis of Heteroaromatic PU_1–5_ Derivatives

##### General Polymerization Process

In a nitrogen-gas-saturated system, 0.002 mol of M2 were dissolved in 30–40 mL of dry pyridine, and 0.002 mol of various aromatic and aliphatic diisocyanates were added dropwise. The flask had three necks, and the condenser was attached. For 18 h, the mixture was warmed at a low simmer. After letting the reaction mixture settle at ambient temperature, it was placed into ice water to precipitate a white-brown substance (PU_1_–PU_5_). The process was completed after the solid polymers were separated, filtered, and finally washed in water [30,31,32]. The polymer product was then dried for two days at 70 °C at low pressure (1 mmHg) [45]. The IR spectra of all produced polymers showed absorption bands at 3300 cm^−1^ (NH of urea derivative) and 1635 cm^−1^ (C=O of urea derivative) as exhibited.

### 2.4. Antimicrobial Screening

Antimicrobial screening of the synthesized polyurea derivatives PU_1_–PU_5_ was performed against different bacterial and fungal organisms. Bacterial cell suspensions were prepared from cultures grown in sterile water on nutritional agar for 48 h [46,47]. One milliliter of cell suspension and fifteen milliliters of NA were placed into a Petri dish with a 9 cm diameter. We gently shook the plate to combine the inoculum. Both the tested polymer solution and the ampicillin solution (0.1 and 0.05 mg/mL in DMSO) (Whatman) were impregnated onto sterile 5 mm filter paper discs. The solvent-impregnated discs were used in conjunction with a control group (DMSO). After drying for 1 h, the impregnated discs were put in the middle of each plate. The seeded plates were incubated for 24–48 h at 30 ± 2 °C. The triplicate sets’ inhibition zone radii (millimeter) were measured, and the findings are shown in later.

#### 2.4.1. Antibacterial Screening

To test the antibacterial activities of the target polyurea, four bacterial species representing both Gram-negative and Gram-positive strains were used: *Escherichia coli* (*E. coli*) and *Pseudomonas aeruginosa* (*P. aeruginosa*) were the representative Gram-negative strains, and *Bacillus cereus* (*B. cereus*) and *Bacillus subtilis* (*B. subtilis*) were the representative Gram-positive strains. To create the cell suspensions, 48-h-old cultures were cultivated in sterile water on nutrient agar. A 9 cm diameter Petri dish was seeded with 1 mL of cell suspension; then, 15 mL of NA was added. The dish was gently shaken to mix the inoculum. We impregnated sterile 5 mm filter paper discs (Whatman) with solutions of the polymer sample under test and ampicillin solution (0.1 and 0.05 mg/mL in DMSO) as a standard. Several discs were also treated with the solvent to serve as controls (DMSO). After drying for 1 h, the impregnated discs were placed in the center of each plate. The seeded plates were incubated for 36–48 h at 30 ± 2 °C. The triplicate sets’ inhibition zone radii (millimeter) were measured, and the results are shown in shown later.

#### 2.4.2. Antifungal Screening

Two important pathogenic fugal organisms were used in this work: *Fausarium oxysporum* (*F. oxysporum*) and *Candida albicans* (*C. albicans*). Using 2- to 5-day-old cultures of the test fungi grown on potato dextrose agar or sabouraud agar medium (SDA), a spore suspension in sterile water was made [46,47]. The subsequently produced spore concentration was 5 × 10^5^ spores/mL. A sterile Petri plate of 9 cm in diameter was filled with 15 mL of the growth media and injected with 1 mL of the spore suspension. To homogenize the inoculum, the plate was gently shaken. The antifungal activity of the polymers was determined using the standard agar disc diffusion method, which is described as follows: The test polymer and dermatin solutions (0.1 or 0.05 mg/mL in DMSO) were impregnated into sterile 5 mm filter paper discs (Whatman). In addition, control discs containing the solvent (DMSO) were employed. Once the impregnated discs had dried for an hour, they were placed in the center of each plate. The plates were seeded and then incubated for 5 days at 30 ± 2 °C. Measurements of the inhibition zone radii (in millimeters) were taken at regular intervals during the incubation period. Using duplicate sets, we were able to observe statistically significant differences between treatments (shown later).

### 2.5. Docking Measurements

Molecular docking is a category of bioinformatics modeling that concerns inducing the interaction of two or more molecules to provide a stable adduct. Then, depending on the binding properties of ligand and target, it is used to predict three-dimensional structures of any degree of complexity. All molecular docking protocols were performed using the MOE 2019.0120 software by employing the triangle matcher method, and refinement was performed using rigid protein and flexible compounds. The docking score and RMSD were determined for the ten highest docking positions by London dG, and the five best scores were obtained. The crystal structures of *1KNZ*, a protein of the Gram-negative bacteria *E. coli*; *1JIJ*, a protein of the Gram-positive bacteria *S. aureus*; and *1IYL*, a protein of *C. albicans*, whose sources are commonly used as model organisms for fungal pathogens, were downloaded from the Protein Data Bank “https://www.rcsb.org/” (accessed on 20 February 2023) [48,49,50,51]. All proteins were isolated and corrected after removing all solvent molecules and cocrystalline compounds. The active site for all proteins was chosen as the exact site of the downloaded structures. The validation of the docking protocol was performed by executing the protocol for a cocrystalline compound, whose RMSD was 1.93 Å (<3.00 Å).

## 3. Results and Discussion

Several potential uses for the studied heteroaromatic thiazole-based polyurea derivatives have been explored. As a result, we used the polycondensation approach to create four novel series of polymers with thiazole rings and sulfur links in their polymer backbones. The novel polymers’ structures were revealed using standard characterization methods. The antimicrobial properties of the produced polymers were also evaluated.

### 3.1. Chemistry and Characterization Tools

**Figure 1:**
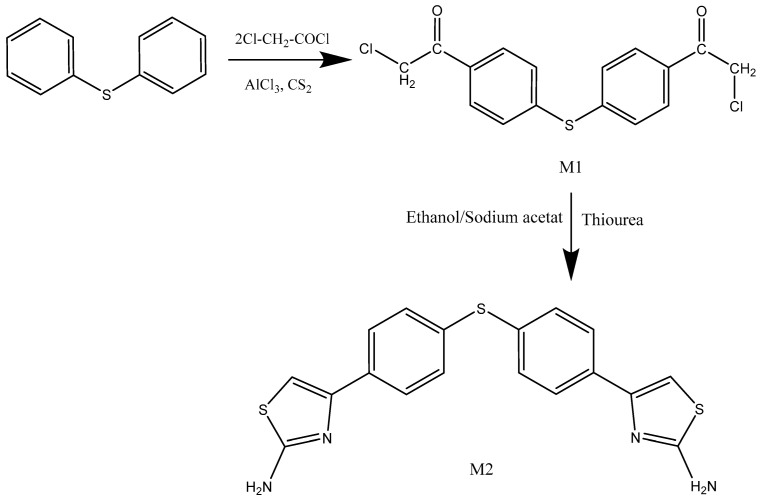
Synthesis of bis-4-chloroacetyl-diphenylsulfide M1 and 2-aminothiazolediphenylsulfide M2 monomers.

First, chloroacetyl chloride and diphenyl sulfide were reacted in dry carbon disulfide with the aid of aluminum chloride to produce 4-bis-chloroacetyl-diphenylsulfide (M1). After 6 h of refluxing M1 and thiourea in 100% ethanol, sodium acetate was added to the resulting reaction mixture to produce the monomer 4-bis-2-aminothiazole diphenyl sulfide (M2) (Figure 1). Melting point measurements were performed on the synthesized monomers, and the findings were found to be in accordance with the published literature [43,44]. Many spectroscopic investigations, including those employing Fourier transform infrared spectroscopy (FT-IR) and proton nuclear magnetic resonance (NMR) spectroscopy (^1^H- and ^13^C-NMR), were conducted to verify the hypothesized structures, as reported in the Experimental section and the Supplemental Information file.

**Figure 2:**
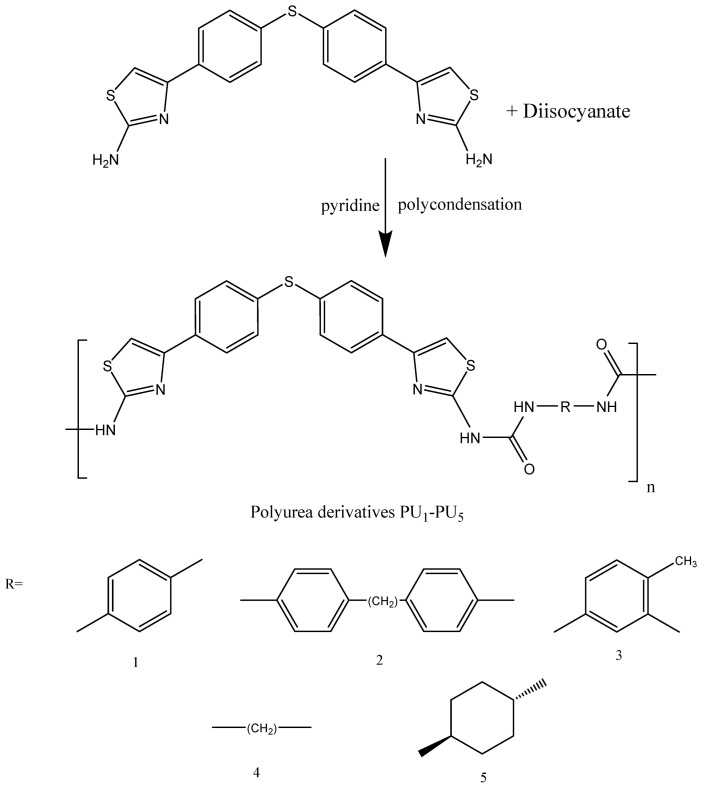
Synthesis of polyurea PU_1_–PU_5_.

Afterward, a new series of polyurea derivatives—PU_1_, PU_2_, PU_3_, PU_4_, and PU_5_—was synthesized using the solution polycondensation procedure through the interaction between M2 and different aromatic aliphatic and cyclic diisocyanates in pyridine, as presented in Figure 2. The synthesis of polyurea linear polymers is generally based on the condensation of diisocyanates with dicarboxylic acid chlorides in dry pyridine [30,31,32].

**Figure 3:**
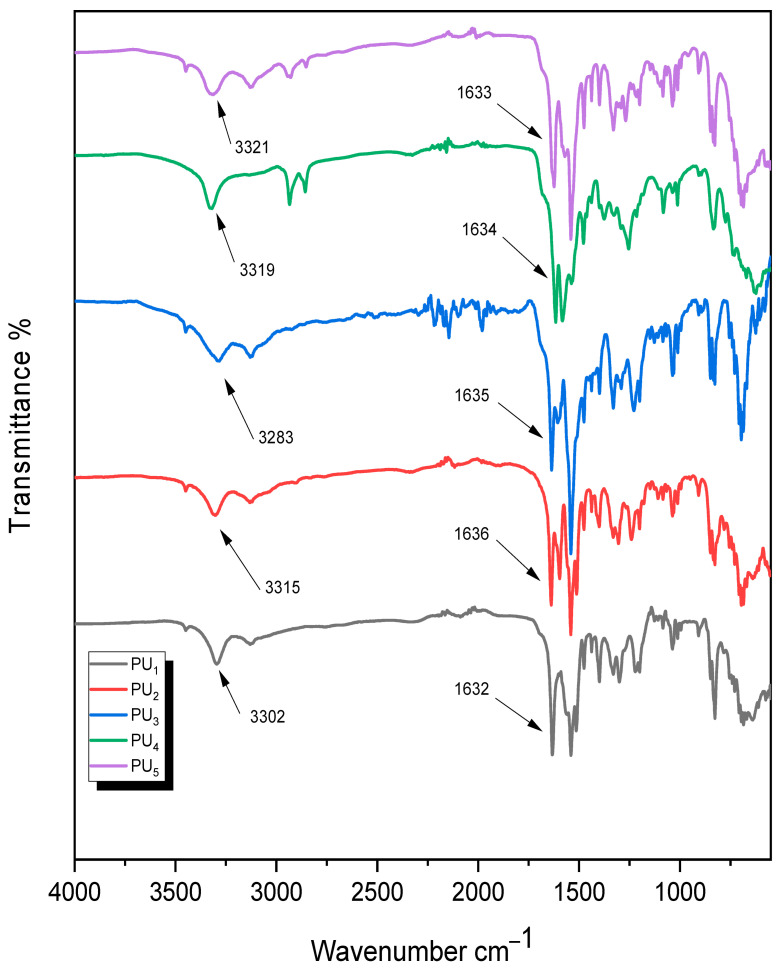
FT.IR spectra of polyurea PU_1_–PU_5_.

The chemical structures of these new polymers were determined using FT-IR analysis, as presented in the Experimental Procedures section. The IR spectra of all the polymers showed absorption bands at 3300 cm^−1^ (NH of urea derivative) and 1635 cm^−1^ (C=O of urea derivative) in addition to the most common characteristic peaks presented in the polymers’ main chains, as illustrated in Figure 3.

The new polymers were also characterized using different standard methods, including a solubility test, GPC molecular weight determinations, X-ray diffraction analysis, thermal analysis, and scanning electron microscopy. The solubility of PU_1_, PU_2_, PU_3_, PU_4_, and PU_5_ was examined at room temperature using many solvents, including CHCl_3_, CH_2_Cl_2_, benzene, acetone, dimethylformamide (DMF), tetrahydrofuran (THF), dimethyl sulfoxide (DMSO), formic acid, and sulfuric acid A 5% (w = v). All the polyurea derivative solutions were prepared under the same conditions and were fully soluble in THF and concentrated H_2_SO_4_, yielding a dark red color, but they were only partially soluble in other aprotic organic solvents such as formic acid, DMF, DMSO, DCM, and chloroform, while in common organic solvents such as benzene and acetone, they were insoluble. Table 1 presents the solubility characteristics of the synthesized polyurea derivatives in various solvents.

The primary technique used to examine molecular weight is gel permeation chromatography. In this study, the GPC values of the studied substances were recorded and calculated by a computer program. The values of the average numbers, weight-average molecular weights, and polydispersity indexes (Mw, Mn, Pw, and DPI) of the polyurea derivatives were determined, and their data are presented in Table 2. In this table, it can be seen that the average molecular weights (Mw) for the tested polymers are nearly in the same range, from 36,629.54 to 43,356.72, which demonstrates that all the resulting polymers have the same chain length [52]. The longest polymer chain was PU_2_, presenting a Pw ≈ 69 and a PDI = 1.08; however, the lowest molecular weight was that of PU_1_, presenting a Pw ≈ 68 and a PDI = 1.13.

**Figure 4:**
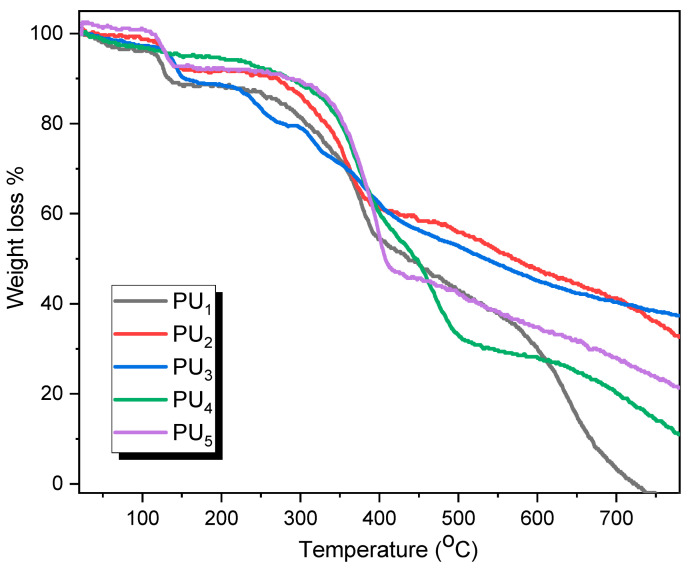
TGA curves of PU_1_–PU_5_ in airflow at a heating rate of 10 °C/min.

The resulting polyurea derivatives were characterized using XRD and TGA to determine their crystallinity and thermal stability, respectively. The data regarding the thermogravimetric analysis of the polyurea derivatives are shown in Table 3 and Figure 4. Table 3 shows the various temperatures for various percentage weight losses. All samples were heated to 800 °C at a rate of 10 °C/min in N_2_, which resulted in the same decomposition curve for all samples with multistep processes, starting with the conformable removal of the (OH) group due to the removal of absorbed moisture and attached solvents that cause weight loss; however, this step starts at room temperature and ends at approximately 105 °C for PU_1_, PU_2_, PU_3_, PU_4_, and PU_5_, with mass losses of 3.8, 1.4, 2.9, 3.4, and 0.1 mg, respectively. The thermographs also show that the polyurea derivatives decompose in three stages. The first one, between 105 °C and 160 °C, is the partial decomposition of all polymers. The second stage starts at 160 °C and ends at 400, 389, 410, 500, and 447 °C for PU_1_, PU_2_, PU_3_, PU_4_, and PU_5_, respectively. In the third stage, degradation becomes maximal at around 550 °C and is nearly complete at around 800 °C. Thus, the new polyurea derivatives exhibit good thermal stability, which may be attributed to the presence of the thiazole moiety and sulfur linkage in the main chain of all new polymers. The initial decomposition temperature (*IDT*) refers to the temperatures at which decomposition starts, while (*FDT*) is defined as the final decomposition temperature and refers to the temperatures at which decomposition is completed [53]. Both values can easily be determined from the TGA curves. All polymers have the same *IDT* (150 ± 2 °C) except PU_1_, which showed a lower IDT value (135 °C), whereas the aromatic-based derivatives (PU_1_–PU_3_) showed higher *FDT* values (616, 655, and 665 °C) compared to the aliphatic-based derivatives’ (PU_4_ and PU_5_) values (590, 605 °C). This observation is attributed to the higher rigidity of the aromatic moieties in the polymers’ main chains compared to the more flexible spacers presented in the aliphatic derivatives [31,32]. Furthermore, the maximum decomposition temperature (*PDT_max_*) refers to the temperature at which decomposition reaches its maximum [54]. The *PDT_max_* values were determined from the corresponding DTG curves. The *PDT_max_* for PU4 and PU5 showed the highest values (430 and 425 °C, respectively) compared to the other derivatives, while PU1 showed the lowest *PDT_max_* value (383 °C). The order of *PDT_max_* obtained values from lowest to highest is as follows: PU_1_ < PU_2_ < PU_3_ < PU_5_ < PU_4_.

**Figure 5:**
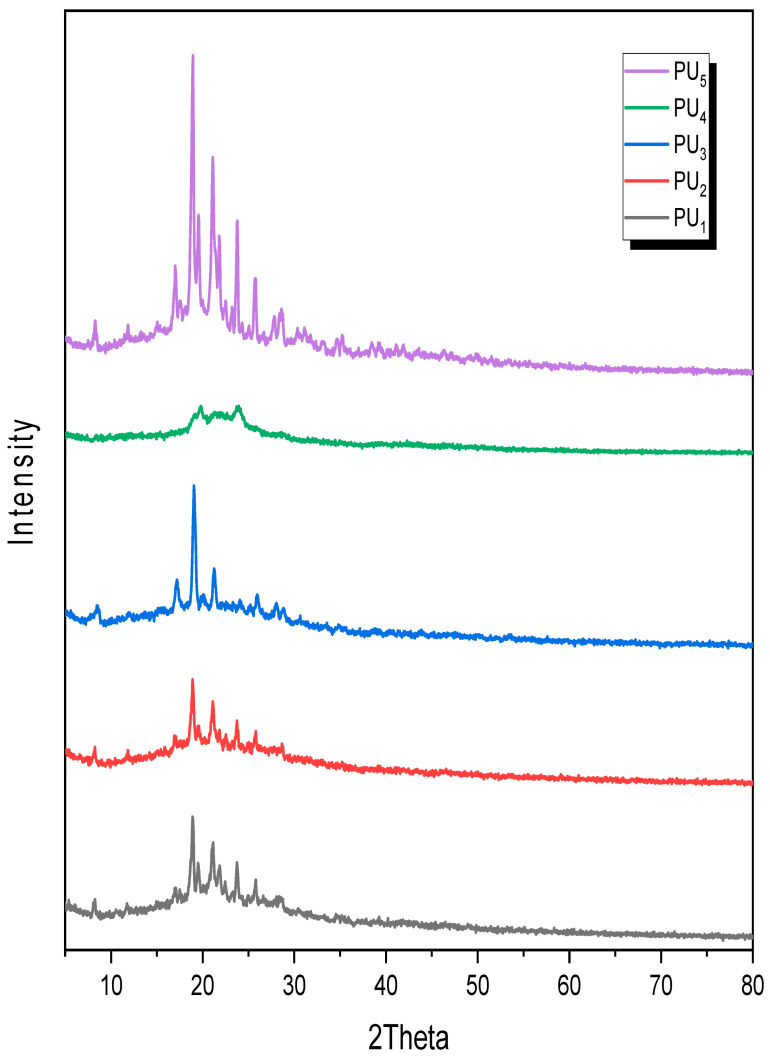
X-ray diffraction patterns of PU_1_, PU_2_, PU_3_, PU_4_, and PU_5_.

Furthermore, the X-ray diffraction patterns of the polyurea derivatives were measured, as shown in Figure 5. The data were acquired over the range of 2*θ* = 5 to 80°, which indicates a high degree of crystallinity for all polymers, except for PU_4_, which exhibited an amorphous halo pattern, possibly because of the six methylene groups, which might be the result of increasing polyurea chain flexibility in the adjacent chains [32].

PU_5_ is the most crystalline of the PUs and can be categorized as a crystalline polymer due to its crystalline diffractogram. The X-ray diffractograms show a large number of reflection peaks that are intermediate between crystalline and amorphous interferences in the same region, indicating the presence of C=O and S as polar groups and high C=C bond levels in the polymers’ main chains, which induce a significant degree of order between the two adjacent chains of polymers, leading to a noticeable increase in crystallinity [43]. In addition, the presence of a high number of C=C bands and C=O bands, which represent polar groups arranged between the adjacent polyurea chains, could have caused this increased crystallinity [32]. 

**Figure 6:**
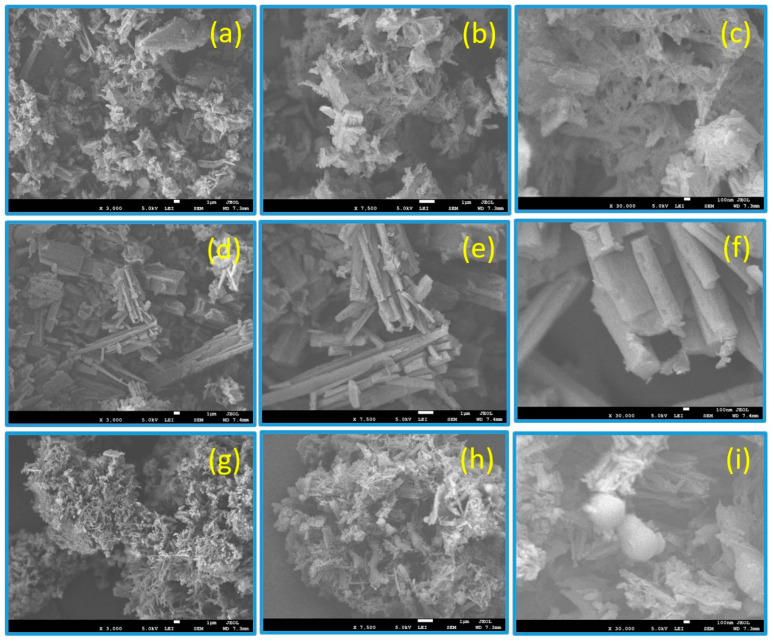
SEM images of PU_1_ (**a**–**c**), PU_4_ (**d**–**f**), and PU_5_ (**g**–**i**) at variable magnifications.

The morphological features of the new polyurea derivatives were studied via SEM measurements, as illustrated in Figure 6. PU_1_, PU_4_, and PU_5_ were employed as the measured samples, indicating that the surface of PU_1_ consisted of micro-holes, yielding spongy, porous shapes at different magnifications (x = 3000, 7500, and 30,000), as illustrated in Figure 6a–c, respectively. Meanwhile, PU_4′_s surface displayed shapes resembling wooden planks, sticks, or rod-like particles at magnifications of x = 3000 (Figure 6e) and 7500 (Figure 6e). The same features appeared at a magnification of x = 30,000 (Figure 6f). The average rod diameter ranged from 100 to 150 nm. Furthermore, the surface of PU_5_ showed coral-reef-like shapes with flowery shapes and noticeable round particles at lower and higher magnifications of x = 3000, 7500, and 30,000 (Figure 6d–f). Such globular particles were clearly visible when zooming in, as illustrated in Figure 6i.

### 3.2. Antimicrobial Screening

Biologically active polymers and/or their related nanocomposites are of significant interest to a huge number of researchers worldwide [55,56,57,58,59,60,61,62,63]. *E. coli* and *P. aeruginosa* were identified as representative Gram-negative bacteria and *B. cereus* and *B. subtilis* were chosen as representative Gram-positive bacteria for the purpose of the antimicrobial screening of all the synthetically produced polyurea derivatives (PU_1_–PU_5_). In order to evaluate the produced polymers’ antifungal properties, a number of different species of fungi, including *F. oxysporum* and *C. albicans*, were utilized. The inhibitory area was measured in millimeters, and the antibacterial and antifungal activities were evaluated in relation to the standard medications Ampicillin and Dermatin, which served as references for the antibacterial and antifungal activities, respectively. 

**Figure 7:**
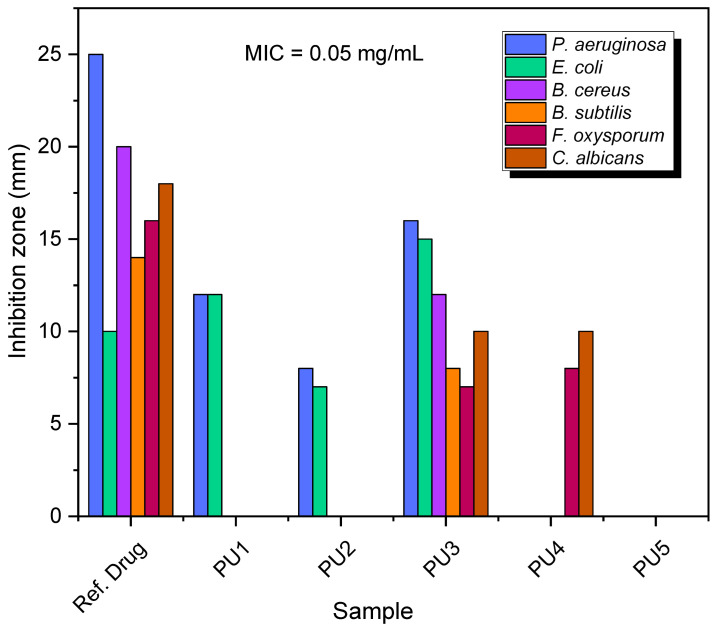
The growth of bacterial and fungi species against PU_1_–PU_5_ at a concentration equal 0.05 (mg/mL).

**Figure 8:**
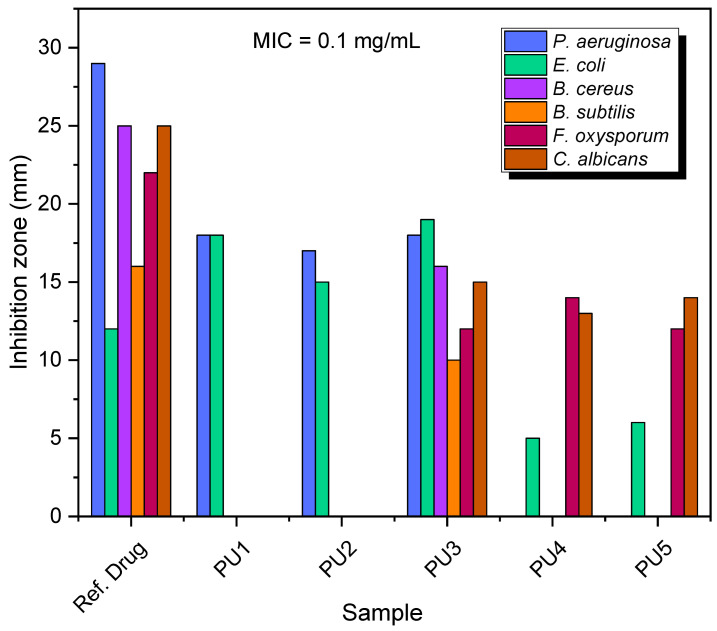
The growth of bacterial and fungi species against PU_1_–PU_5_ at a concentration equal to 0.1 (mg/mL).

Figure 7 and Figure 8 contain illustrations of all of the results from the antimicrobial screening of the synthesized polymers at two different concentrations: 0.05 and 0.1 (mg/mL). The findings presented in Table 4 demonstrate that the examined compounds showed varying levels of antibacterial activity.

PU_3_ had the greatest inhibitory impact on the bacteria and fungi studied. The positive controls were able to establish inhibition zones of a significant magnitude against these bacteria and fungi. Both PU_1_ and PU_2_ demonstrated a notable level of antibacterial activity against the employed Gram-negative bacteria (*E. coli* and *P. aeruginosa*). In addition, neither *C. albicans* nor *A. flavus* were susceptible to any kind of antifungal activity exhibited by PU_1_ or PU_2_. Both variants produced the same outcomes when tested against the Gram-positive bacteria that were used in the study (*B. cereus* and *B. subtilis*). In contrast with the other compounds, PU_4_ and PU_5_ demonstrated antifungal activities that were on the lower end of the spectrum.

### 3.3. Docking Study

All polyurea derivatives (PU_1–5_) were screened for the presence of the *1KNZ* protein of the Gram-negative bacteria *E. coli*. *1KZN* codes for the 24 kDa gyrase fragment, which is the main protein involved in the replication and transcription of bacterial circular DNA [48,49]. Furthermore, the *1JIJ* protein is contained in the Gram-positive bacteria *S. aureus* [50,51], and the *1IYL* protein of *C. albicans* is commonly used as a model organism for fungal pathogens [64,65].

**Figure 9:**
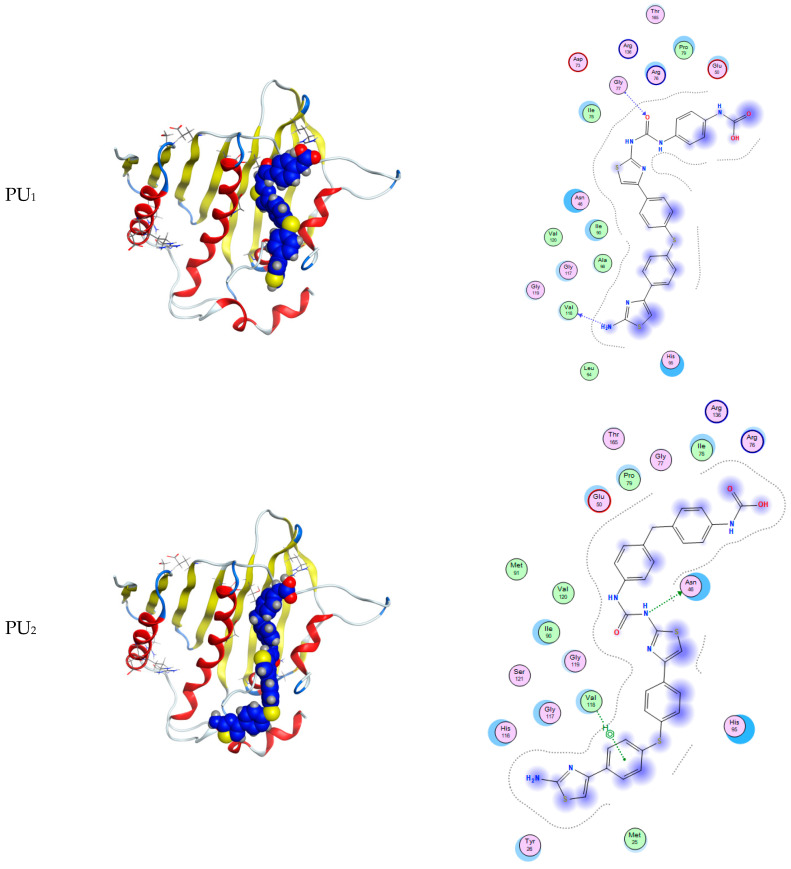

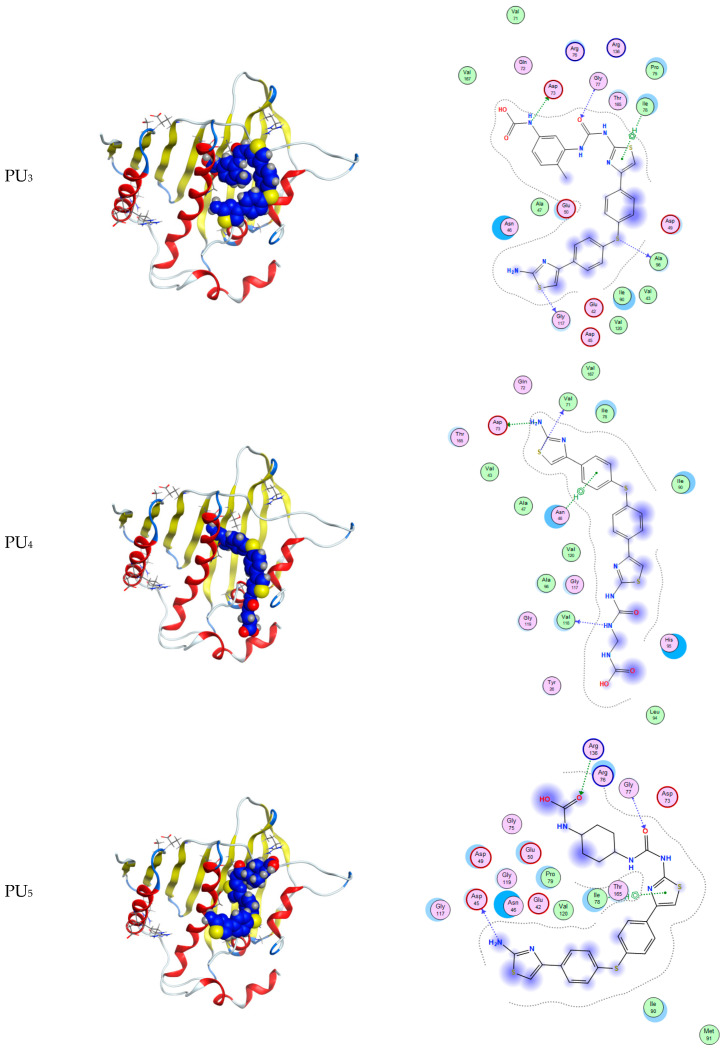

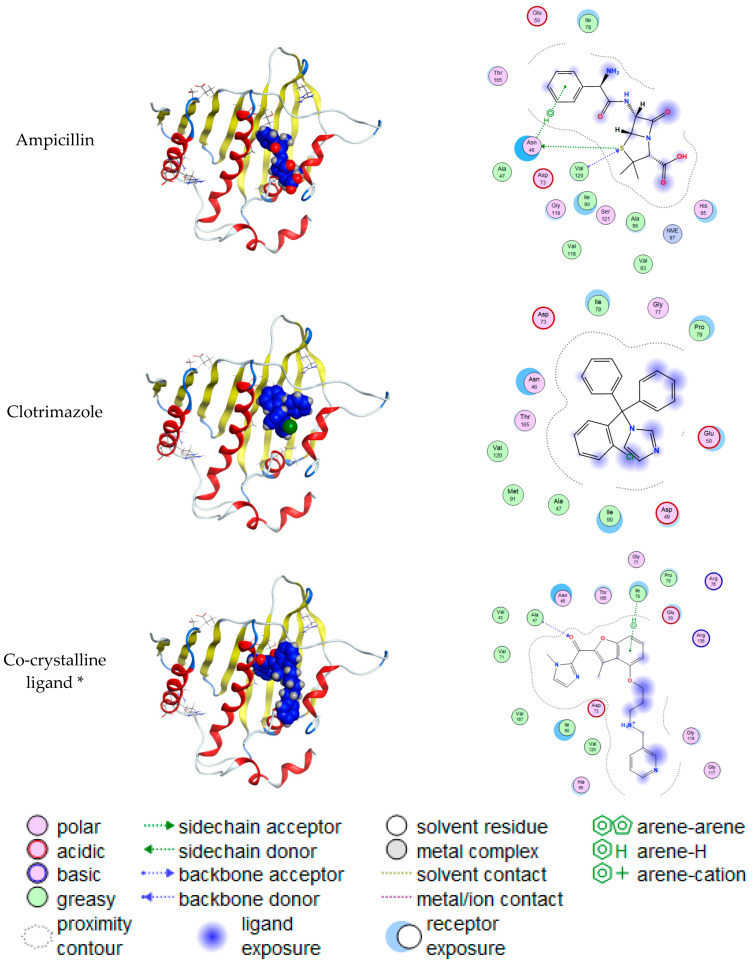
2D and 3D interaction of polyurea derivatives PU_1_–PU_5_ with *1KNZ* protein of *E. coli* bacterial for Gram-negative bacteria. * ([2-AMINO-3-(4-HYDROXY-PHENYL)-PROPIONYLAMINO]-(1,3,4,5-TETRAHYDROXY-4-HYDROXYMETHYL-PIPERIDIN-2-YL)-ACETIC ACID).

**Figure 10:**
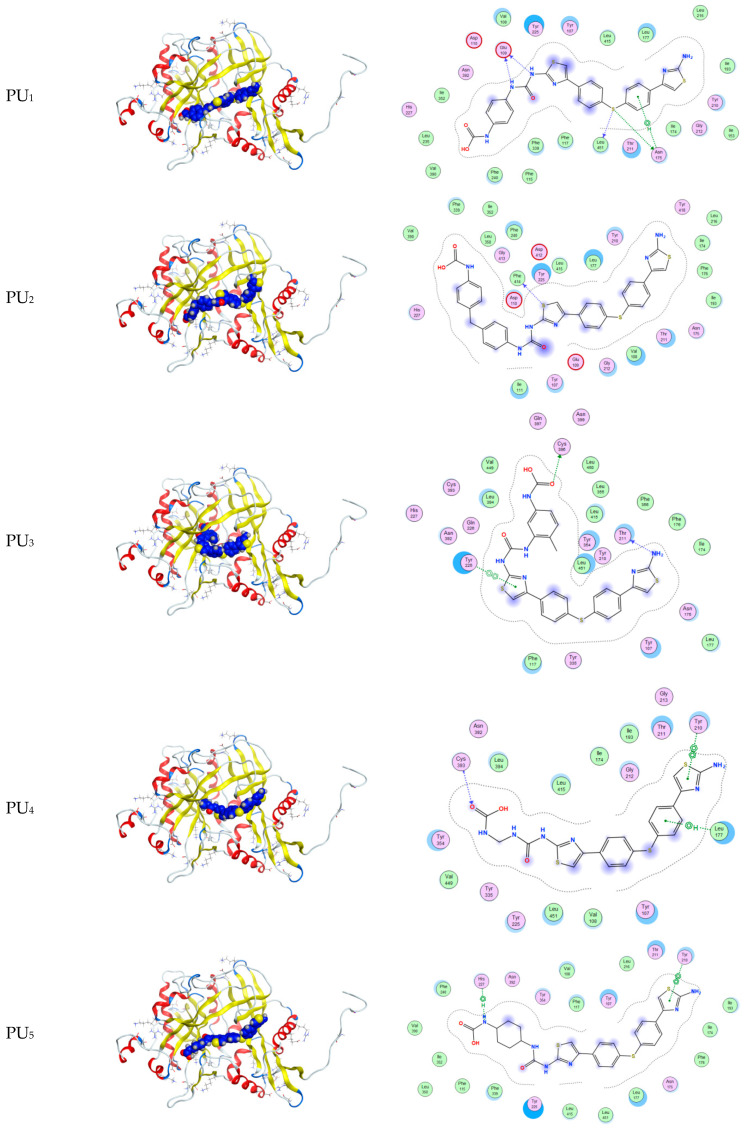

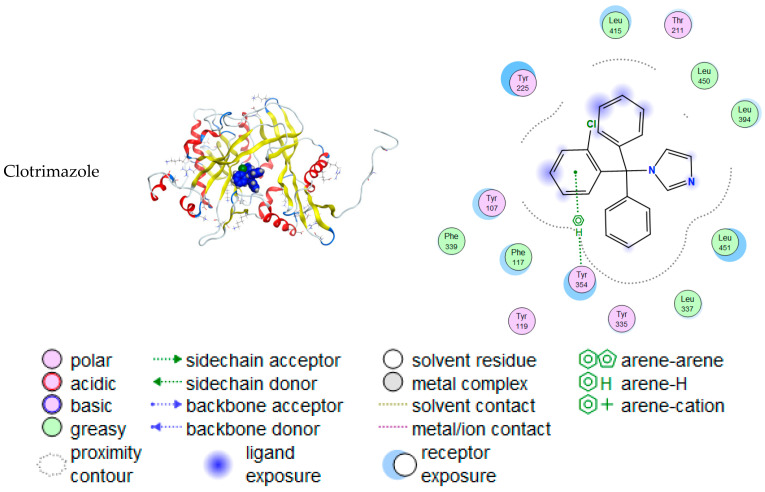
2D and 3D interaction of polyurea derivatives PU_1_–PU_5_ with “*1IYL*” protein for *C. albicans*as (a model organism for fungal pathogens).

The docking scores of the five polymers with the proteins *1KNZ*, *1JIJ*, and *1IYL* are compatible with the experimental data in Figure 9 and Figure 10 as well as Figure S7 (see Supplementary Information file). The cocrystalline ligand 3-((3-methyl-2-(1-methyl-1H imidazole-2-carbonyl)benzofuran-4-yl)oxy)-N-(pyridin-3-ylmethyl)propan-1-aminium was redocked; the RMSD value of this compound was 1.85 Å with the *1KNZ* protein, and its docking score was −6.72 k.Cal (Table S1).

PU_3_ has the highest docking score of all the Gram-positive, Gram-negative, and fungal proteins among all other compounds, with docking scores of −9.97, −9.04, and −10.55 k.Cal, respectively (Table 5 and Table 6 and Table S2 (see Supporting Information file)). These results are in agreement with the obtained experimental results against the selected bacteria and fungi.

For the Gram-negative protein, the most effective compounds were PU_1_, PU_2_, and PU_3_, which have a greater degree of aromaticity in their structures than the other two compounds, i.e., PU_4_ and PU_5_ (Table 7). With regard to the docking result for the Gram-positive protein, the only compound with high activity is PU_3_, presenting −9.04 kcal/mol of activity via two hydrogen-π stacking interactions of 2.43 and 2.82 Å (Table S3 (see Supporting Information file)). This finding is in line with the findings of the experiments conducted against the selected bacteria and fungi.

The docking results for all compounds against *the 1IYL* protein of *C. albicans*, which is commonly used as a model organism for fungal pathogens, show that compounds PU_3_, PU_4_, and PU_5_ have the best results among the remaining compounds and the highest docking scores among all other proteins, presenting docking scores of −10.55, −10.38, and −10.26 k.Cal, respectively (Table 8).

## 4. Conclusions

By performing solution polycondensation of the monomer M2 with five distinct aromatic, aliphatic, and cyclic diisocyanates in pyridine, a new family of sulfur-containing heteroaromatic polyurea derivatives based on thiazole moieties coupled with thioether linkages was produced with high yields. The structures of the new polymers were confirmed using XRD, TGA, and SEM and, subsequently, characterized using FT-IR spectroscopy. In thermal stability tests (TGA), all the polymers performed well. The obtained XRD data confirm that PU_5_ displays the highest crystallinity, whereas PU_4_ displays the lowest. Matching DTG curves were used to calculate the *PDT_max_* values, of which those for PU_4_ and PU_5_ derivatives were the highest (430 and 425 °C, respectively) when compared to the other derivatives. Meanwhile, PU_1_ had the lowest *PDT_max_* (383 °C). Aside from PU_1_, all the polymers had the same IDT (15 ± 2 °C), but PU_1_ had a lower IDT value (135 °C). The surface of PU_1_ was made up of micro-holes that created spongy, porous shapes, whereas the surface of PU_4_ displayed shapes resembling wooden planks and sticks. Moreover, the surface of polyurea PU_5_ showed a morphology resembling coral reefs with flowery shapes at different magnifications. Both PU_1_ and PU_2_ demonstrated a notable level of antibacterial activity against the studied Gram-negative bacteria (*E. coli* and *P. aeruginosa*). Furthermore, three distinct proteins were used in 2D and 3D molecular docking investigations, with the results correlating with those of the antimicrobial screening. PU_3_ had the highest docking score with all Gram-positive, Gram-negative, and fungal proteins among all other compounds, with docking scores of −9.97, −9.04, and −10.55 k.Cal, respectively.

## Figures and Tables

**Table 1:** Solubility characteristics of PU_1_, PU_2_, PU_3_, PU_4_, and PU_5_.

Polymer Code	THF	DMF	HCOOH	CHCl_3_	CH_2_Cl_2_	DMSO	H_2_SO_4_	Benzene	Acetone
PU_1_	+	+	+ −	+ −	+ −	+	+	−	−
PU_2_	+	+	+ −	+ −	+ −	+	+	−	−
PU_3_	+	+	+	+ −	+ −	+	+	−	−
PU_4_	+	+	+	+ −	+ −	+	+	−	−
PU_5_	+	+	+	+ −	+ −	+	+	−	−

+ indicates solubility at room temperature. + − indicates partial solubility. − indicates insolubility.

**Table 2:** The GPC results for PU_1_, PU_2_, PU_3_, PU_4_, and PU_5_.

Sample	Formula	GPC Results			
		^a^ Mw	^b^ Mn	^c^ Pw	PDI
PU_1_	C_26_H_18_O_2_S_3_N_6_	36,629.54	32,278.21	~68	1.13
PU_2_	C_33_H_24_O_2_S_3_N_6_	43,356.72	39,982.38	~69	1.08
PU_3_	C_27_H_20_O_2_S_3_N_6_	42,318.73	37,762.15	~76	1.12
PU_4_	C_26_H_26_O_2_S_3_N_6_	40,752.82	36,676.33	~74	1.11
PU_5_	C_26_H_24_O_2_S_3_N_6_	38,562.90	32,224.77	~70	1.20

^a^ Weight-average molecular weight, ^b^ number-average molecular weight, and ^c^ average number of repeating units.

**Table 3:** Thermal properties of polymers PU_1_, PU_2_, PU_3_, PU_4_, and PU_5_.

Polymer Code	*IDT* ^a^	*PDT_max_* ^b^	*FDT* ^a^	Temperature (°C) at the Indicated Weight Loss Level ^a^				
				10%	20%	30%	40%	50%
PU_1_	135	383	616	131.1	306.4	357.5	381.5	437.4
PU_2_	151	392	655	237.6	329.7	361.6	422	566
PU_3_	153	420	665	154.6	276	356	411.4	528
PU_4_	150	430	590	281.8	351	372	398.7	446
PU_5_	148	425	605	295	355	375	393.7	406.7

^a^ Values determined via TGA at a heating rate of 10 °C min^−1^; ^b^ values determined via DTG.

**Table 4:** Antimicrobial screening of sulfur-based polyurea PU_1_–PU_5_.

Bacterial and Fungi Species	MIC (mg/mL)/Inhibition Zone (mm)										
	PU1		PU2		PU3		PU4		PU5	REF. DRUG *	
	0.05	0.1	0.05	0.1	0.05	0.1	0.05	0.1	0.1	0.05	0.1
*P. aeruginosa (−ve)*	12	18	8	17	16	18	-	-	-	25	29
*E. coli (−ve)*	12	18	7	15	15	19	-	5	6	10	12
*B. cereus (+ve)*	-	-	-	-	12	16	-	-	-	20	25
*B. subtilis (+ve)*	-	-	-	-	8	10	-	-	-	14	16
*F. oxysporum*	-	-	-	-	7	12	8	14	12	16	22
*C. albicans*	-	-	-	-	10	15	10	13	14	18	25

* Reference drugs: Ampicillin for antibacterial and Dermatin for antifungal.

**Table 5:** Docking score of polyurea derivatives PU_1_–PU_5_ with *1KNZ* protein of the bacteria *E. coli*, which was used as a representative Gram-negative bacterium.

Compound	S	rmsd_refine	E_conf	E_place	E_score1	E_refine	E_score2
PU_1_	−8.32	2.82	−188.54	−71.04	−9.56	−43.50	−8.32
	−7.10	1.83	−199.04	−54.83	−10.00	−42.54	−7.10
	−6.96	3.35	−194.20	−60.43	−9.89	−42.94	−6.96
	−6.84	1.97	−192.40	−36.39	−9.70	−37.04	−6.84
	−6.83	3.43	−195.25	−52.34	−9.78	−35.56	−6.83
PU_2_	−8.18	2.68	−175.11	−40.71	−8.54	−46.30	−8.18
	−8.15	1.49	−177.01	−76.26	−11.76	−49.99	−8.15
	−7.57	2.04	−176.19	−52.13	−11.08	−43.61	−7.57
	−7.45	1.29	−186.73	−71.18	−9.39	−45.11	−7.45
	−7.44	1.37	−180.86	−40.86	−10.28	−44.85	−7.44
PU_3_	−9.97	2.78	−195.85	−60.48	−9.58	−44.43	−9.97
	−7.34	2.90	−195.46	−52.64	−9.32	−40.95	−7.34
	−7.22	1.50	−199.60	−37.13	−9.76	−43.08	−7.22
	−7.12	1.71	−183.44	−60.21	−9.48	−41.99	−7.12
	−6.89	1.59	−196.45	−76.74	−9.56	−42.30	−6.89
PU_4_	−7.12	1.86	−254.20	−52.79	−10.08	−42.79	−7.12
	−6.83	1.49	−260.67	−71.55	−9.81	−40.67	−6.83
	−6.70	3.00	−251.28	−74.48	−9.72	−37.24	−6.70
	−6.55	3.25	−243.10	−65.57	−9.67	−35.80	−6.55
	−6.47	3.22	−248.20	−69.48	−9.40	−37.27	−6.47
PU_5_	−7.32	1.44	−214.28	−64.42	−9.91	−39.68	−7.32
	−7.16	3.12	−225.18	−76.59	−9.39	−41.82	−7.16
	−7.00	1.89	−218.32	−59.40	−9.35	−37.23	−7.00
	−6.98	1.50	−215.39	−32.51	−10.32	−38.15	−6.98
	−6.95	3.82	−226.98	−71.83	−9.44	−36.21	−6.95
Ampicillin	−5.91	1.78	71.46	−68.89	−10.26	−28.31	−5.91
	−5.86	1.70	71.25	−113.25	−10.75	−28.46	−5.86
	−5.68	1.52	70.18	−77.40	−9.44	−28.92	−5.68
	−5.65	1.63	68.26	−57.00	−9.50	−25.64	−5.65
	−5.63	2.18	69.90	−58.44	−9.73	−29.62	−5.63
Coocrystaline ligand *	−6.72	1.89	63.05	−51.27	−10.74	−36.22	−6.72
	−6.56	1.13	68.88	−64.48	−12.99	−35.57	−6.56
	−6.45	2.65	68.67	−58.49	−10.66	−37.31	−6.45
	−6.40	2.18	73.84	−73.95	−11.39	−36.90	−6.40
	−6.31	1.79	62.77	−56.91	−9.62	−37.48	−6.31

* ([2-AMINO-3-(4-HYDROXY-PHENYL)-PROPIONYLAMINO]-(1,3,4,5-TETRAHYDROXY-4-HYDROXYMETHYL-PIPERIDIN-2-YL)-ACETIC ACID).

**Table 6:** Docking interaction of compounds PU_1_–PU_5_ with “*1IYL*” protein for *C. albicans*, which was used as a model organism for fungal pathogens.

Compound	Ligand	Receptor	Interaction	Distance	E (kcal/mol)
PU_1_	S 17	OD1 ASN 175 (A)	H-donor	3.17	−1.0
	S 17	O LEU 451 (A)	H-donor	3.78	−0.8
	N 34	O GLU 109 (A)	H-donor	2.86	−1.0
	N 37	O GLU 109 (A)	H-donor	3.17	−0.6
	6-ring	CA ASN 175 (A)	pi-H	4.45	−1.1
PU_2_	S 31	O PHE 414 (A)	H-donor	3.45	−0.8
PU_3_	O 43	SG CYS 396 (A)	H-donor	3.32	−2.2
	N 59	O THR 211 (A)	H-donor	2.89	−0.6
	5-ring	6-ring TYR 225 (A)	pi-pi	3.75	−0.0
PU_4_	O 43	CA CYS 393 (A)	H-acceptor	3.52	−0.5
	6-ring	CD2 LEU 177 (A)	pi-H	3.58	−0.5
	5-ring	6-ring TYR 210 (A)	pi-pi	3.77	−0.0
PU_5_	N 40	5-ring HIS 227 (A)	H-pi	4.54	−0.6
	5-ring	6-ring TYR 210 (A)	pi-pi	3.87	−0.0
Clotrimazole	6-ring	CE2 TYR 354 (A)	pi-H	3.57	−0.5

**Table 7:** Docking interaction of polyurea derivatives PU_1_–PU_5_ with *1KNZ* protein of the bacteria *E. coli* for Gram-negative bacteria.

Compound	Ligand	Receptor	Interaction	Distance	E (kcal/mol)
PU_1_	N 56	O VAL 118 (A)	H-donor	3.27	−0.8
	O 39	N GLY 77 (A)	H-acceptor	3.09	−0.8
PU_2_	N 34	OD1 ASN 46 (A)	H-donor	2.88	−1.6
	6-ring	CG2 VAL 118 (A)	pi-H	3.85	−0.7
PU_3_	S 14	O GLY 117 (A)	H-donor	3.42	−0.6
	S 17	O ALA 96 (A)	H-donor	3.74	−0.6
	N 40	OD1 ASP 73 (A)	H-donor	2.94	−3.2
	O 39	N GLY 77 (A)	H-acceptor	3.14	−0.8
	5-ring	CA ILE 78 (A)	pi-H	4.55	−0.6
PU_4_	S 14	O VAL 71 (A)	H-donor	3.38	−0.8
	N 37	O VAL 118 (A)	H-donor	3.31	−0.7
	N 49	OD1 ASP 73 (A)	H-donor	3.14	−0.6
	6-ring	CB ASN 46 (A)	pi-H	3.76	−0.5
PU_5_	N 62	O ASP 45 (A)	H-donor	3.22	−0.9
	O 39	N GLY 77 (A)	H-acceptor	3.00	−2.2
	O 43	NH1 ARG 136 (A)	H-acceptor	3.41	−1.8
	5-ring	CD1 ILE 78 (A)	pi-H	3.60	−0.5
Ampicillin	S 11	OD1 ASN 46 (A)	H-donor	3.53	−1.2
	S 11	N VAL 120 (A)	H-acceptor	3.68	−2.3
	6-ring	CB ASN 46 (A)	pi-H	3.62	−1.0

**Table 8:** Docking scores of polyurea derivatives PU_1_–PU_5_ with “*1IYL*” protein for *C. albicans*, which was used as a model organism for fungal pathogens.

Compound	S	rmsd_refine	E_conf	E_place	E_score1	E_refine	E_score2
PU_1_	−6.10	1.09	−197.38	−103.01	−11.60	−52.02	−9.10
	−8.96	1.53	−191.40	−110.64	−12.14	−50.77	−8.96
	−8.77	1.79	−190.53	−91.83	−12.14	−51.93	−8.77
	−8.65	1.50	−187.76	−103.18	−11.67	−49.78	−8.65
	−8.50	2.81	−192.08	−82.72	−11.95	−52.62	−8.50
PU_2_	−6.19	2.01	−171.61	−92.87	−11.06	−54.28	−10.19
	−9.07	2.65	−188.81	−110.51	−11.59	−65.98	−10.07
	−9.77	1.63	−178.53	−74.76	−12.48	−57.47	−9.77
	−9.66	2.04	−179.01	−111.53	−11.82	−56.45	−9.66
	−9.65	1.66	−164.10	−113.76	−10.99	−37.83	−9.65
PU_3_	−10.55	1.39	−181.92	−88.39	−15.12	−26.83	−10.55
	−8.74	1.32	−179.45	−75.81	−11.13	−52.64	−8.74
	−8.71	1.83	−186.38	−99.22	−11.17	−49.58	−8.71
	−8.69	1.55	−185.70	−86.65	−11.10	−39.86	−8.69
	−8.57	1.63	−192.73	−110.09	−11.35	−51.56	−8.57
PU_4_	−10.38	1.93	−252.28	−81.21	−11.41	−43.02	−8.38
	−8.27	1.98	−256.21	−88.77	−11.48	−47.00	−8.27
	−8.21	1.07	−253.17	−115.55	−12.21	−37.40	−8.21
	−8.19	2.18	−253.32	−72.08	−13.30	−44.80	−8.19
	−8.07	1.27	−256.19	−104.55	−11.64	−41.54	−8.07
PU_5_	−10.26	2.98	−209.10	−112.46	−14.04	−47.36	−9.26
	−9.19	1.34	−221.04	−111.48	−12.28	−52.10	−9.19
	−9.18	1.10	−221.12	−128.05	−12.61	−52.65	−9.18
	−9.17	1.20	−202.99	−129.44	−12.53	−51.57	−9.17
	−9.03	1.82	−209.09	−105.05	−11.83	−48.56	−9.03
Clotrimazole	−10.24	0.83	101.50	−81.28	−9.28	−23.71	−6.24
	−6.04	1.97	110.81	−54.06	−8.87	−21.56	−6.04
	−5.95	1.61	103.67	−69.99	−8.57	−21.93	−5.95
	−5.94	1.73	113.61	−74.56	−8.89	−22.11	−5.94
	−5.87	1.59	112.77	−61.83	−8.73	−20.78	−5.87

## Data Availability

Data presented in this study are available on request from the corresponding author.

## Supplementary Materials

The following supporting information can be downloaded at: https://www.mdpi.com/article/10.3390/polym15122662/s1, Figure S1. I.R spectrum of bis-4-chloroacetyl-diphenylsulfide; Figure S2. ^1^HNMR spectrum of chloroacetyl-diphenylsulfide; Figure S3. ^13^CNMR spectrum of chloroacetyl-diphenylsulfide; Figure S4. I.R spectrum of 2-aminothiazole diphenylsulfide; Figure S5. ^1^HNMR spectra of 2-aminothiazol diphenylsulfide+ with D_2_O; Figure S6. ^13^CNMR spectrum of 2-aminothiazol diphenylsulfide; Figure S7. 2D and 3D interaction of polyurea derivatives PU_1_–PU_5_ with “*1JIJ*” protein fo-r *Staphy-lococcus aureus* as positive-Gram bacteria; Table S1. Validation of docking method with gentamycin and co-crystalline compound with “*1JIJ*” protein for *Staphylococcus aureus* as positive-Gram bacteria; Table S2. Docking score of polyurea derivatives PU_1_–PU_5_ with “*1JIJ* protein for *Staphylococcus aureus* as positive-Gram bacteria. Table S3. Docking interaction of polyurea derivatives PU_1_–PU_5_ with “*1JIJ*” protein for Staphylococcus aureus as positive-Gram bacteria.
