# Supplementary material for: Favorable Heteroaromatic Thiazole-Based Polyurea Derivatives as Interesting Biologically Active Products
Source: Polymers (Basel). 2023 Jun 13;15(12):2662. doi: 10.3390/polym15122662 (PMC10303549; doi:10.3390/polym15122662)
Supplement: Supplementary file 1 [file polymers-15-02662-s001.zip › polymers-2370127-supplementary.pdf]

Table S1. Validation of docking method with gentamycin and co-crystalline compound with *"1JIJ"* protein for *Staphylococcus aureus* as positive-Gram bacteria.

Validation compound	Docking Score	rmsd_refine	E_conf	E_place	E_score1	E_refine	E_score2
Co-Crystalline ligand *	−6.72	1.89	63.05	−51.27	−10.74	−36.22	−6.72
	−6.56	1.13	68.88	−64.48	−12.99	−35.57	−6.56
	−6.45	3.65	68.67	−58.49	−10.66	−37.31	−6.45
	−6.40	2.18	73.84	−73.95	−11.39	−36.90	−6.40
	−6.31	1.79	62.77	−56.91	−9.62	−37.48	−6.31

*3-((3-methyl-2-(1-methyl-1H-imidazole-2-carbonyl)benzofuran-4-yl)oxy)-N-(pyridin-3-ylmethyl)propan-1-aminium.

Table S2. Docking score of polyurea derivatives PU_1–PU_5 with *"1JIJ"*protein for *Staphylococcus aureus* as positive-Gram bacteria.

	S	rmsd_refine	E_conf	E_place	E_score1	E_refine	E_score2
PU1	8.07	2.01	−191.34	−76.96	−12.46	−59.11	−8.07
	−8.01	1.94	−187.96	−103.66	−12.69	−54.59	−8.01
	−7.57	2.41	−182.02	−105.11	−13.57	−50.18	−7.57
	−7.53	1.82	−186.62	−114.40	−12.02	−53.32	−7.53
	−7.49	1.51	−183.65	−120.05	−15.69	−56.73	−7.49
PU2	−8.06	1.92	−182.67	−79.14	−11.09	−55.47	−8.57
	−8.04	2.26	−182.24	−99.70	−13.43	−52.54	−8.04
	−8.02	2.71	−163.52	−80.54	−14.15	−44.26	−8.02
	−8.02	1.55	−179.31	−114.96	−12.46	−54.62	−8.02
	−8.01	2.05	−171.54	−42.57	−10.68	−54.10	−8.01
PU3	−9.04	1.50	−197.76	−104.59	−12.03	−55.61	−9.04
	−8.86	3.10	−192.23	−80.95	−11.40	−56.05	−8.86
	−8.76	1.46	−193.05	−122.04	−12.89	−52.91	−8.76
	−8.73	1.41	−197.32	−107.59	−11.38	−56.22	−8.73
	−8.71	2.13	−181.81	−98.73	−13.36	−50.32	−8.71
PU4	−8.48	1.29	−248.67	−94.90	−12.82	−44.03	−8.48
	−8.01	1.50	−249.10	−90.09	−13.87	−52.84	−8.01
	−8.01	1.52	−249.11	−87.38	−11.40	−43.34	−8.01
	−7.91	3.30	−249.99	−64.16	−11.82	−45.17	−7.91
	−7.89	1.36	−252.97	−81.95	−11.36	−52.65	−7.89
PU5	−7.83	1.38	−213.00	−85.17	−12.54	−41.73	−8.83
	−7.68	2.12	−216.21	−136.08	−13.36	−53.87	−8.68
	−7.66	2.10	−208.56	−58.30	−11.63	−48.28	−8.66
	−7.64	1.50	−219.33	−79.82	−11.43	−53.54	−8.64
	−7.57	1.74	−227.72	−99.85	−11.43	−50.72	−8.57
Ampicillin	−7.09	2.85	75.28	−74.94	−11.17	−38.44	−7.09
	−7.06	1.12	83.85	−72.27	−11.98	−30.04	−7.06
	−7.04	1.40	77.94	−70.05	−11.89	−28.08	−7.04
	−7.02	1.83	70.63	−95.75	−11.39	−39.01	−7.02
	−6.96	2.26	81.25	−74.87	−12.51	−29.86	−6.96

Table S3. Docking interaction of polyurea derivatives PU_1-PU_5 with *"1JIJ"* protein for *Staphylococcus aureus* as positive-Gram bacteria.

Compound	Ligand	Receptor	Interaction	Distance	E (kcal/mol)
**PU1**	O 39	SG CYS 37 (A)	H-donor	3.53	−0.9
	O 44	OD1 ASP 177 (A)	H-donor	2.89	−3.3
	O 39	N GLY 193 (A)	H-acceptor	3.06	−2.1
**PU2**	S 31	O PRO 222 (A)	H-donor	3.65	−1.4
	6-ring	CA LYS 231 (A)	pi-H	3.76	−0.6
**PU3**	6-ring	CA GLY 233 (A)	pi-H	4.43	−0.5
	5-ring	CB LYS 234 (A)	pi-H	3.82	−1.1
**PU4**	S 14	O PRO 222 (A)	H-donor	3.24	−0.6
	N 49	O PRO 222 (A)	H-donor	3.24	−1.2
**PU5**	O 39	SG CYS 37 (A)	H-donor	3.26	−1.9
Ampicillin	C 12	OD2 ASP 80 (A)	H-donor	3.38	−0.5
	O 15	N ASP 40 (A)	H-acceptor	3.24	−2.0
	C 40	5-ring HIS 50 (A)	H-pi	4.42	−0.7

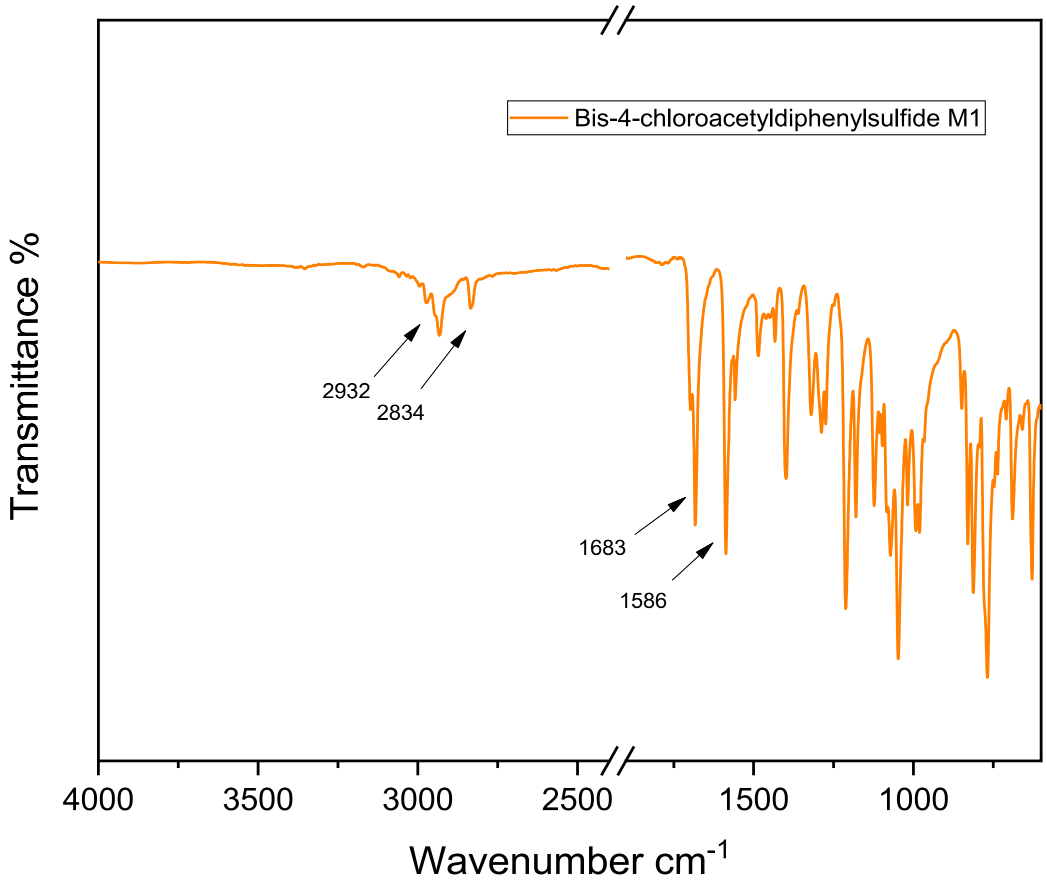

Figure S1. I.R spectrum of bis-4-chloroacetyl-diphenylsulfide.

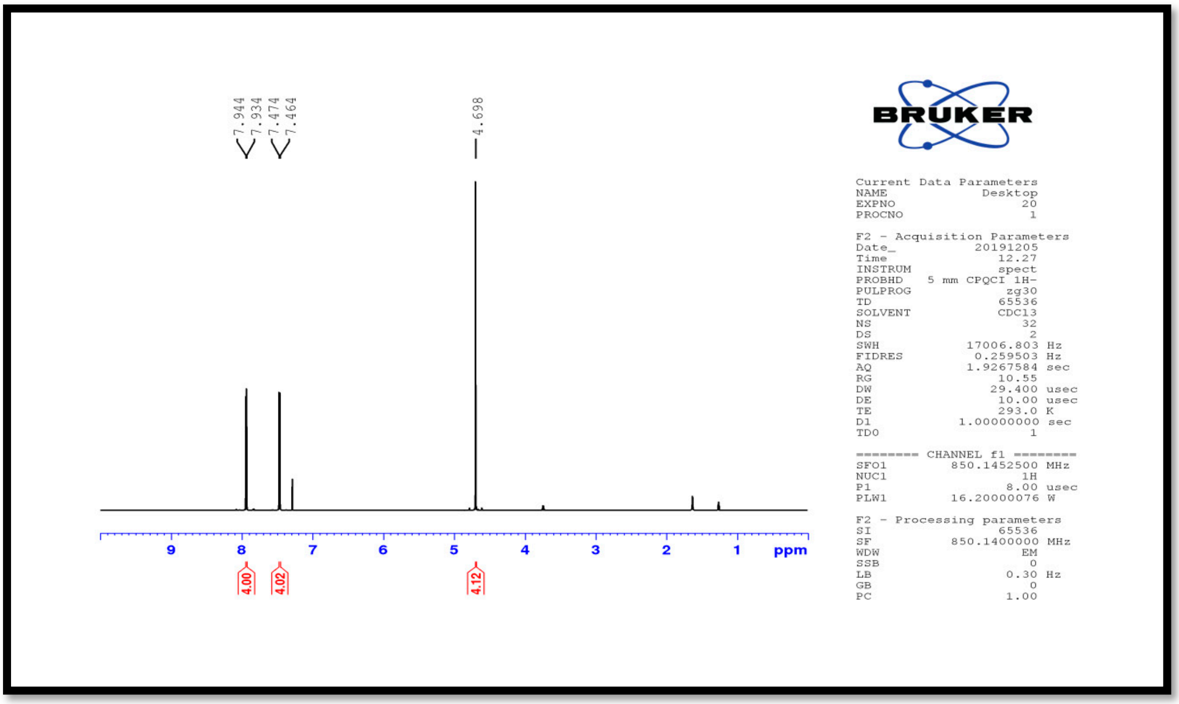

Figure S2. ^{1}HNMR spectrum of chloroacetyl-diphenylsulfide.

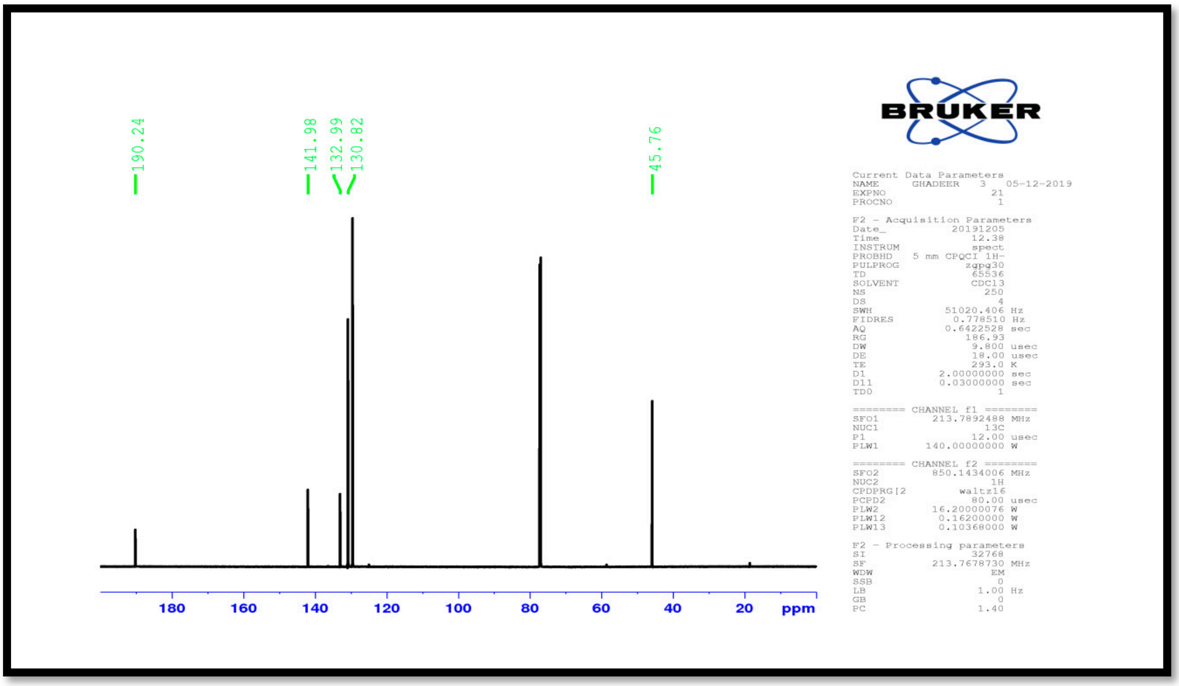

Figure S3. ^{13}CNMR spectrum of chloroacetyl-diphenylsulfide.

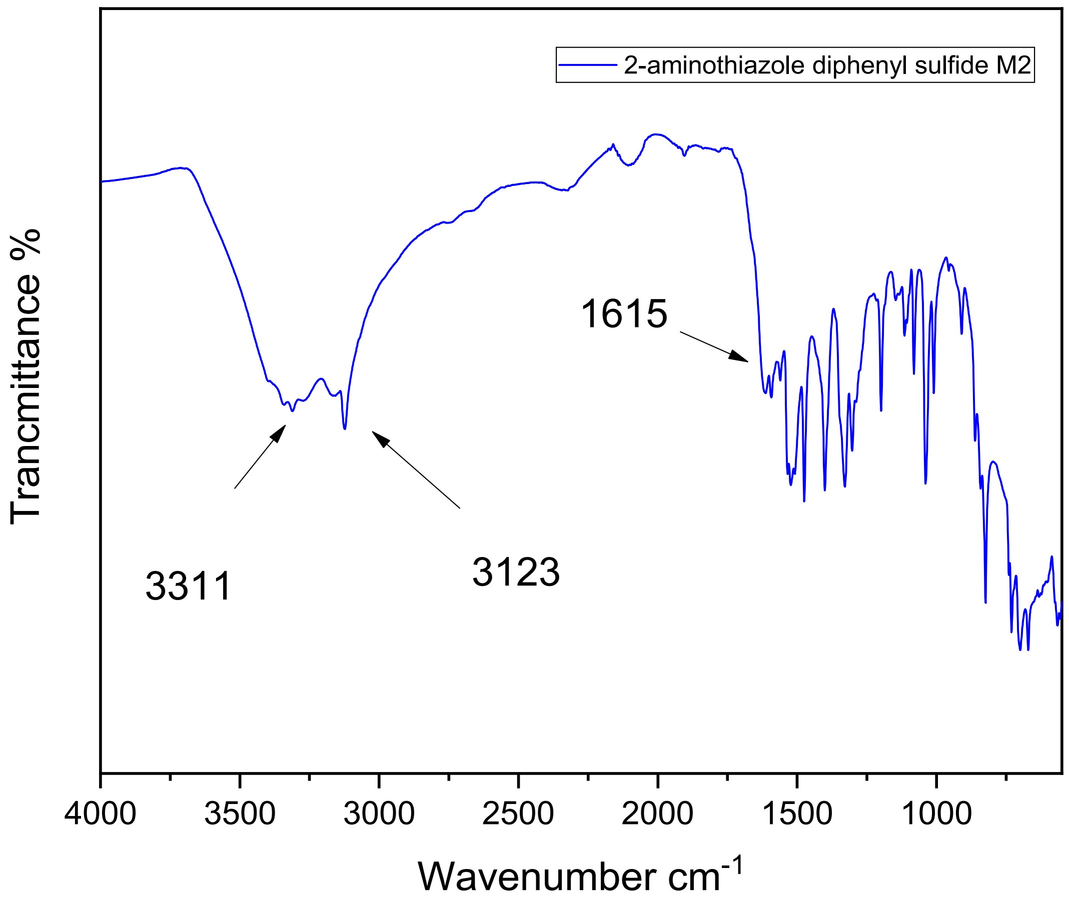

Figure S4. I.R spectrum of 2-aminothiazole diphenylsulfide.

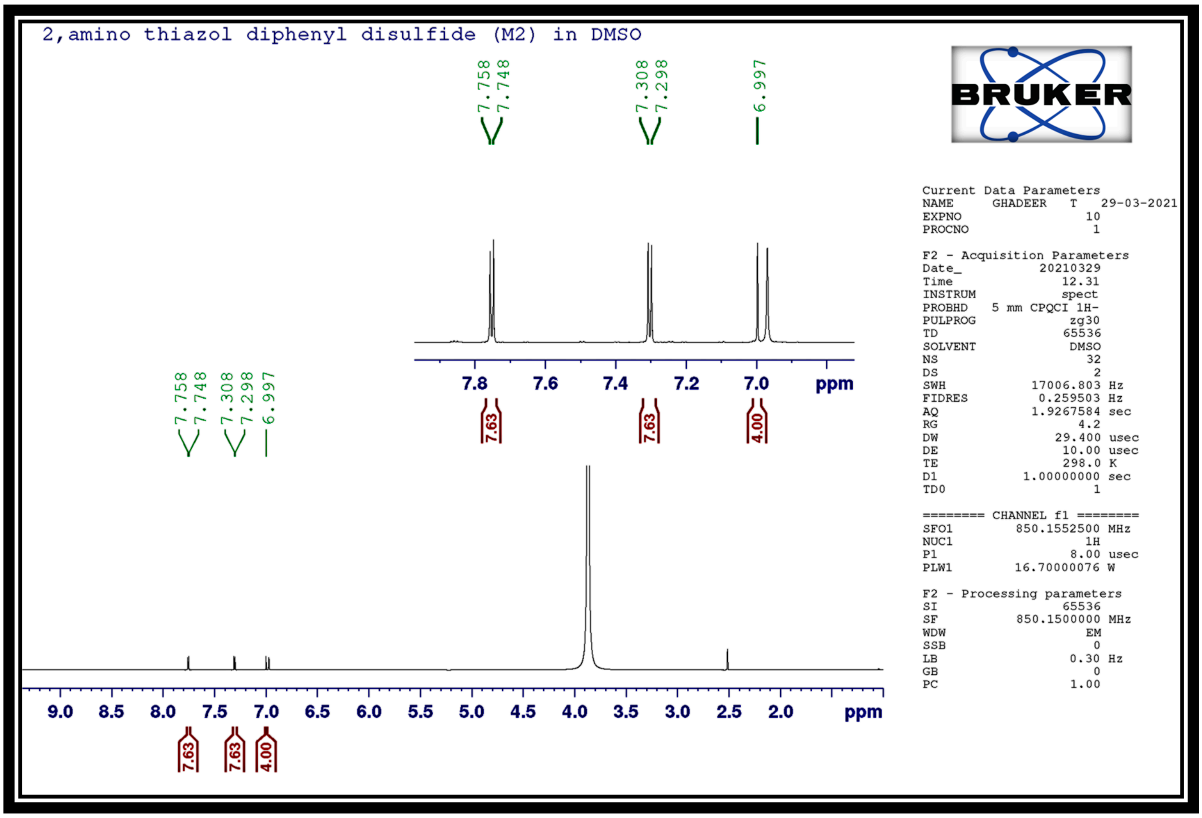

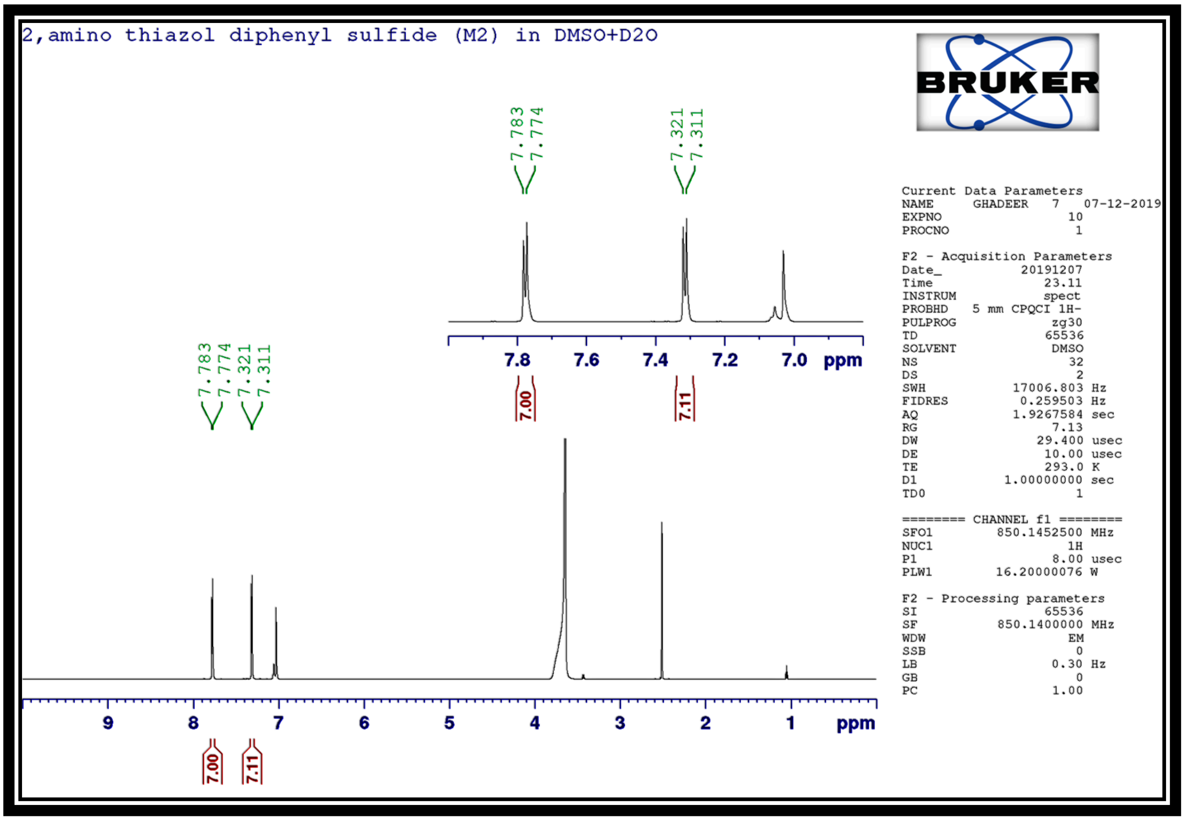

Figure S5. ^{1}HNMR spectra of 2-aminothiazol diphenylsulfide+ with D_2O.

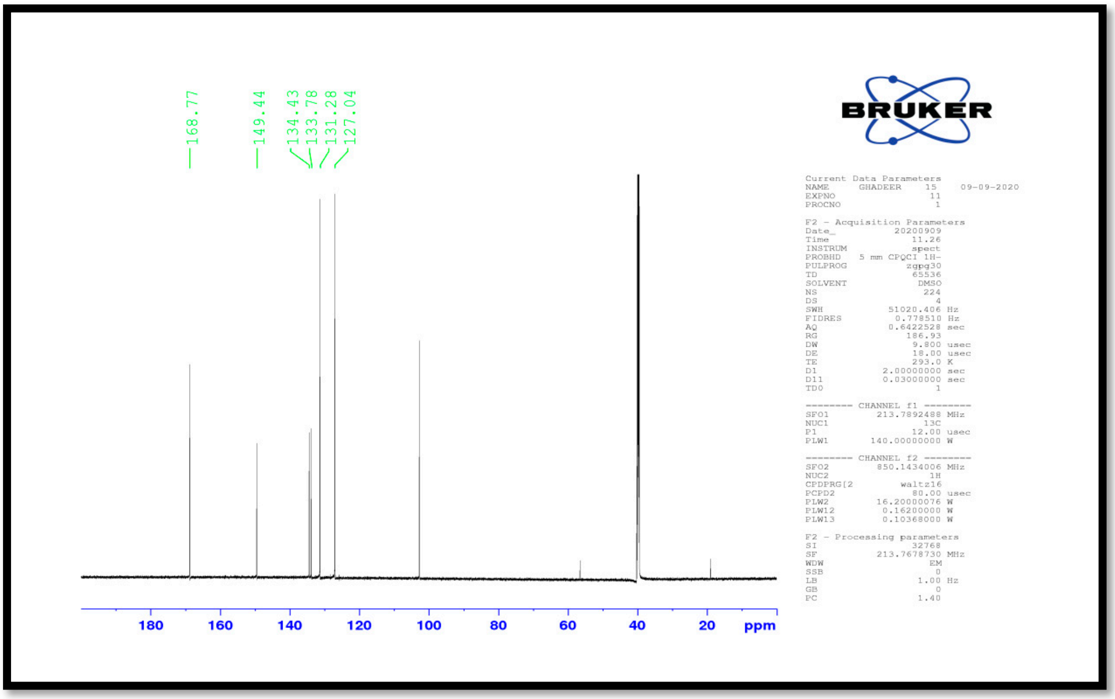

Figure S6. ^{13}CNMR spectrum of 2-aminothiazol diphenylsulfide.

**PU1**	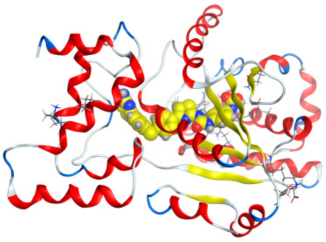	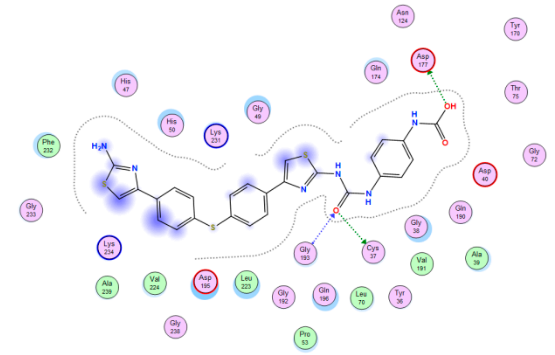
**PU2**	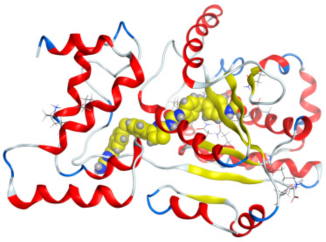	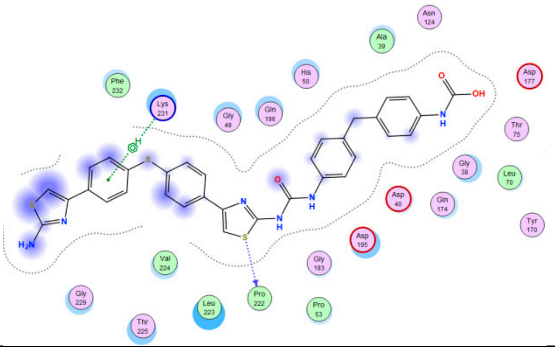
**PU3**	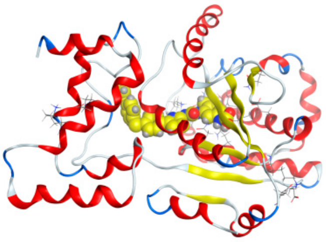	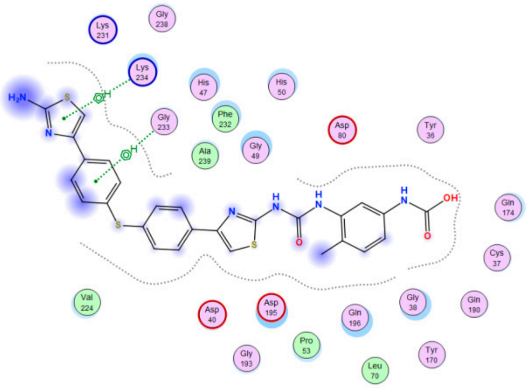
**PU4**	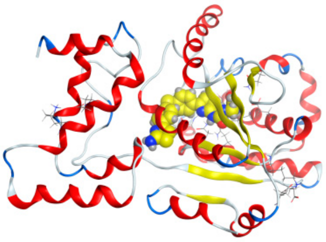	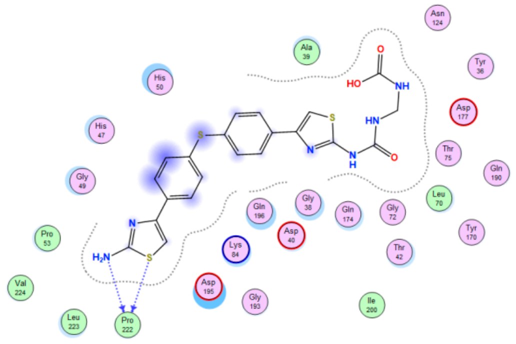
**PU5**	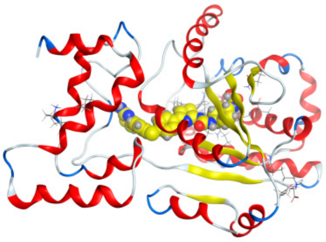	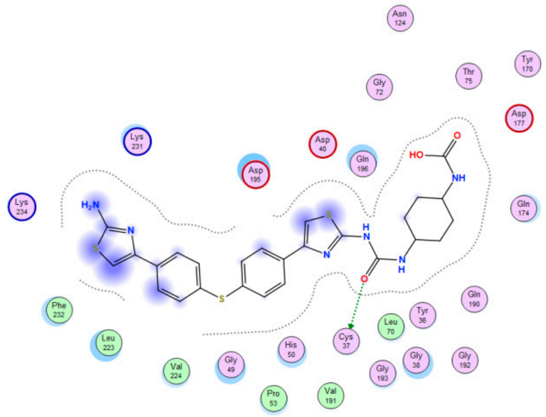

Ampicillin

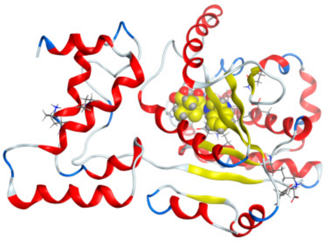

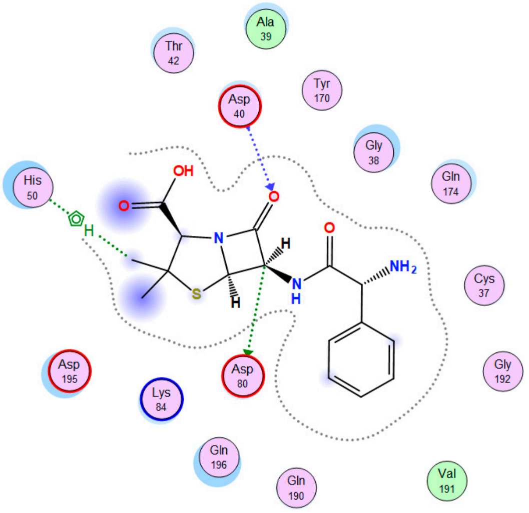

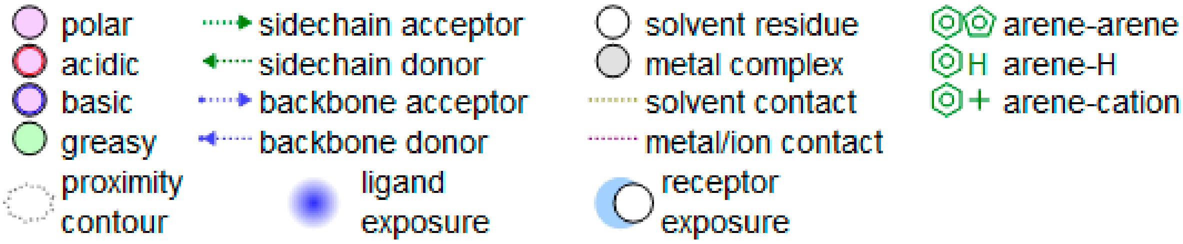

Figure S7. 2D and 3D interaction of polyurea derivatives PU_1–PU_5 with "*1JIJ*" protein fo-r *Staphylococcus aureus* as positive-Gram bacteria.
